# Sanggenol L Induces Apoptosis and Cell Cycle Arrest via Activation of p53 and Suppression of PI3K/Akt/mTOR Signaling in Human Prostate Cancer Cells

**DOI:** 10.3390/nu12020488

**Published:** 2020-02-14

**Authors:** Yeong-Seon Won, Kwon-Il Seo

**Affiliations:** Department of Biotechnology, Dong-A University, Busan 49315, Korea; wonys@dau.ac.kr

**Keywords:** sanggenol L, prostate cancer, apoptosis, AIF, p53, PI3K/Akt/mTOR

## Abstract

Prostate cancer is the most common cancer in Western countries. Recently, Asian countries are being affected by Western habits, which have had an important role in the rapid increase in cancer incidence. Sanggenol L (San L) is a natural flavonoid present in the root barks of *Morus alba*, which induces anti-cancer activities in ovarian cancer cells. However, the molecular and cellular mechanisms of the effects of sanggenol L on human prostate cancer cells have not been elucidated. In this study, we investigated whether sanggenol L exerts anti-cancer activity in human prostate cancer cells via apoptosis and cell cycle arrest. Sanggenol L induced caspase-dependent apoptosis (up-regulation of PARP and Bax or down-regulation of procaspase-3, -8, -9, Bid, and Bcl-2), induction of caspase-independent apoptosis (up-regulation of AIF and Endo G on cytosol), suppression of cell cycle (down-regulation of CDK1/2, CDK4, CDK6, cyclin D1, cyclin E, cyclin A, and cyclin B1 or up-regulation of p53 and p21), and inhibition of PI3K/Akt/mTOR signaling (down-regulation of PI3K, p-Akt, and p-mTOR) in prostate cancer cells. These results suggest the induction of apoptosis via suppression of PI3K/Akt/mTOR signaling and cell cycle arrest via activation of p53 in response to sanggenol L in prostate cancer cells.

## 1. Introduction

Prostate cancer is one of the most common internal cancers in men. Factors such as age, race, stress, and a high-fat diet are reported to be the principal causes of increases in cancer incidence [1]. Nevertheless, Asian countries are reported to have lower incidences of cancers compared to those in Western countries due to the Asian diet of soy, tea, fish, fruits, and vegetables [2,3]. Recently, however, Asian countries are being affected by Western habits, such as the consumption of red meat and a high-fat-high-calorie diet, which have had an important role in the rapid increase in cancer incidence [4]. Effective treatment strategies are required to relieve the social burdens associated with this increase, such as the poor quality of life and increased mortality due to the occurrence of cancer.

Many studies have reported that natural substances have physiological activities, especially anticancer activities [2,5,6]. While there are high risks associated with chemotherapy, radiation therapy, and surgical operations, natural substance treatments are reported to have lower risks as their anticancer properties only target cancer cells [7]. Effective components derived from natural substances such as catechins, curcumin, and genistein have anticancer activities [8,9,10]. For example, auriculacin, celastrol, and decursin have recently been reported to induce cell death via apoptosis in prostate cancer cells [11,12,13].

The leafs and fruit of *Morus alba* (also known as mulberry) has been used as a traditional medicinal treatment and is known to have various effects such as acting as an antihypertensive and hypoglycemic agent with antiedemic, diuretic, and hypolipidemic actions [14,15,16]. It is used not only as a medicinal herb but also as a tea and in food cooking. Recent studies have reported that effective components, such as sanggenol L, sanggenol Q, and sanggenol F, obtained from the root of M. alba possess anti-cancer, anti-inflammatory, and anti-diabetic effects [17]. However, investigations into the physiological and molecular mechanisms of those effective components are still not widely reported.

Sanggenol L (San L) induces cytotoxic and apoptotic activities in ovarian cancer cells via activation of caspases and inhibition of NF-κB signaling [18]. In various cancers, apoptosis is well known as one of the representative molecular and cellular mechanisms associated with natural compounds [11,12,13]. Commonly, caspases play essential roles in programmed cell death, also known as apoptosis [19]. Caspases, when stimulated by internal or external factors, can induce an apoptotic-signaling cascade, which eventually results in apoptotic cell death [19,20]. In addition, apoptosis-inducing factor (AIF), which is released from mitochondria, is involved in the caspase-independent pathway of apoptosis. Mitochondrial permeabilization leads to the release of AIF for participation in DNA degradation during apoptotic cell death [21]. Previous studies have demonstrated that AIF contributes to caspase-independent apoptotic cell death in various cancer cell types [22,23,24,25]. However, whether sanggenol L induces apoptosis in prostate cancer cells via caspase-dependent or caspase-independent pathways has not been examined. Moreover, the apoptotic mechanism of sanggenol L in primary malignant tumor (RC-58T/h/SA#4)-derived human prostate cells has not been described.

DU145, PC-3, and LNCaP are common human prostate cancer cell lines that have been used as in vitro human cell culture models [26]. These cell lines were derived from metastatic sites (brain, bone, and supraclavicular lymph nodes, respectively), whereas the RC-58T/h/SA#4 human prostate cell line was derived from a primary malignant tumor site. Therefore, in this study, we consider that it can reflect the genetic makeup and biological behavior of both primary prostate tumors and metastatic prostate tumors.

In this study, we investigated whether sanggenol L exerts cytotoxic and apoptotic effects in prostate cancer cells via caspase-dependent or caspase-independent pathways. Furthermore, we examined the apoptotic mechanism of sanggenol L in RC-58T/h/SA#4 primary malignant tumor-derived human prostate cells. This study is the first to show that apoptosis and cell cycle arrest in human prostate cancer cells can be induced by sanggenol L via activation of the tumor suppressor p53 and suppression of PI3K/Akt/mTOR signaling.

## 2. Materials and Methods

### 2.1. Chemicals and Reagents

**Figure 1:**
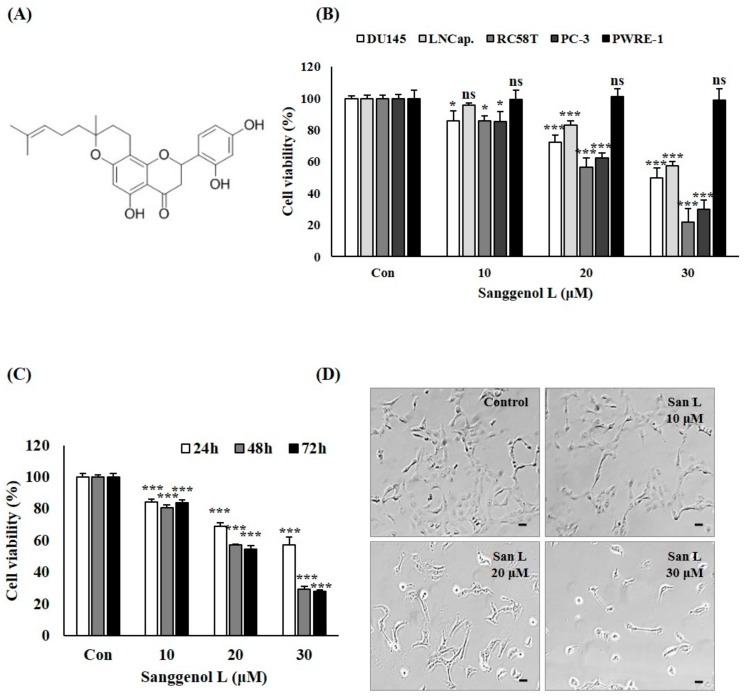
Sanggenol L inhibits cell growth in various human prostate cancer cell lines. (**A**) Chemical structure of sanggenol L. (**B**) Cell viabilities on DU145, LNCap, RC-58T, and PC-3 cells were tested after treatment with or without 10, 20, and 30 µM sanggenol L for 48 h. Cell viability was measured by SRB assay. Results were expressed as the percentage of control. Data values were expressed as mean ± SD of triplicate determinations. Significant differences were compared to the control at * *p* < 0.05 and *** *p* < 0.001 using one-way ANOVA. (**C**) Cell viability of RC-58T cells was tested after treatment with or without 10, 20, and 30 µM sanggenol L for 24, 48, and 72 h by SRB assay. Results were expressed as the percentage of control. Data values were expressed as mean ± SD of triplicate determinations. Significant differences were compared to the control at *** *p* < 0.001 using one-way ANOVA. (**D**) RC-58T cells were treated with or without 10, 20, or 30 µM sanggenol L for 48 h. Cell morphological changes were visualized by inverted microscopy (200×). Scale bar, 100 μm.

Sanggenol L was purchased from Wuhan ChemFaces Biochemical Co., Ltd. (Wuhan, Hubei, China) (Figure 1A). Anti-caspase-3 (sc-7272), anti-caspase-8 (sc-7890), anti-caspase-9 (sc-133109), anti-Bid (sc-514622), anti-Bax (sc-7480), anti-Bcl-2 (sc-7382), anti-poly (ADPribose) polymerase-1 (PARP-1) (sc-56197), anti-AIF (sc-13116), anti-Endonuclease G (Endo G) (sc-365359), anti-CDK1/2 (sc-53219), anti-CDK4 (sc-56277), anti-CDK6 (sc-7961), anti-Cyclin D1 (sc-8396), anti-Cyclin E (sc-247), anti-Cyclin A (sc-239), anti-Cyclin B1 (sc-7393), anti-p53 (sc-126), anti-p21 (sc-6246), anti-PI3K (sc-423), anti-Akt 1/2/3 (sc-8312), anti-p-Akt 1/2/3 (sc-7985-R), anti-mTOR (sc-8319), anti-p-mTOR (sc-101738), and anti-β-actin (sc-47778) antibodies were purchased from Santa Cruz Biotechnology (Santa Cruz, CA, USA). Anti-p-PI3K (4228S) antibodies were purchased from Cell Signaling Technology (Danvers, MA, USA). An ECL kit was purchased from Amersham Life Science (Amersham, UK). Trypsin-EDTA, penicillin, keratinocyte-SFM medium, fetal bovine serum (FBS), antibiotic-antimycotic, and dulbecco’s modified eagle’s medium (DMEM)were purchased from GIBCO BRL Co. (Gaithersburg, MD, USA). Bisbenzimide H 33258 (Hoechst 33258) and sulforhodamin B (SRB) were purchased from Sigma-Aldrich Co. Ltd. (St. Louis, MO, USA). The universal caspase inhibitor (z-VAD-fmk), PI3K inhibitor (LY294002), and AIF inhibitor (N-phenylmaleimide, N-PM) were obtained from R & D Systems (Minneapolis, MN, USA). 

### 2.2. Cell Culture

RC-58T (human prostate cancer derived from a primary malignant tumor site), DU145 (human prostate cancer cells derived from brain), LNCaP-FGC (human prostate cancer cells derived from lymph node), PC-3 (human prostate cancer cells derived from bone), and RWPE-1 (human normal cells derived from prostate epithelium) cells (ATCC, Rockville, ND, USA) were cultured in Keratinocyte-SFM (RWPE-1) or DMEM supplemented with streptomycin (100 μg/mL), penicillin (100 IU/mL), and 10% FBS (Gibco BRL, Life Technologies, Grand Island, NY, USA) in an incubator (5% CO_2_, 37 °C).

### 2.3. Cell Viability Using Sulforhodamin B Assay

In prostate cancer cells, cell viability was examined according to the method of reference study [27]. The RC-58T, DU145, LNCaP-FGC, PC-3, and RWPE-1 cells were seeded at a concentration of 5 × 10^4^ cells/well and incubated with various concentrations of sanggenol L for 48 h. 10% trichloro-acetic acid was added at 4 °C and the plate was washed and air-dried. The cells were stained with 0.4% (*w*/*v*) SRB at room temperature. The bound SRB was solubilized with 10 mM Tris (pH 10.5), after which the absorbance was measured at 540 nm using a microplate reader (Molecular Devices, San Jose, CA, USA).

The influences of LY294002 (a PI3K inhibitor), N-phenylmaleimide (an N-PM and AIF inhibitor) and z-VAD-fmk (a caspases inhibitor) were also demonstrated by using the SRB assay. The RC-58T cells were seeded at a concentration of 5 × 10^4^ cells/well in a 48-well plate, then cultured for 24 h in DMEM. Next, the cells were pre-incubated with 5 μM LY294002, 10 μM N-PM, or 10 μM z-VAD-fmk for 2 h and then treated with sanggenol L (10, 20, and 30 µM) for 48 h. Cell viability was measured at 540 nm using a microplate reader (Molecular Devices, San Jose, CA, USA).

### 2.4. Apoptotic Cell Detection Using Annexin V Staining

Annexin V staining for measuring apoptotic cell death was performed by applying Muse™ Annexin V and dead cell reagent according to the manufacturer’s protocols. the RC-58T cells were seeded at a density of 5 × 10^4^ cells per well in 6-well plates, then cultured for 24 h. Cells were treated with sanggenol L (10, 20, and 30 µM) for 48 h, after which cells were washed twice with 0.001% FBS-PBS buffer. Next, 100 μL of reagent and 10 μL of cell suspension were added to each microcentrifuge tube and the tubes incubated at room temperature for 20 min. Cells were then analyzed using a Muse™ cell analyzer (Merck KGaA, Darmstadt, Germany). The cell percentages were calculated from the mean fluorescence intensity in each of the four quadrants and flow cytometry data were obtained from 5000 events (gated cells) per sample. The coefficient of variation from the mean fluorescence was less than 10%.

### 2.5. DNA Fragmentation

The RC-58T cells were seeded at a concentration of 5 × 10^4^ cells/well and cultured for 24 h. After culturing, the cells were treated with sanggenol L (10, 20, and 30 µM) for 48 h. The cells were subsequently lysed with lysis buffer (10 mM Tris-HCl, pH 7.5, 10 mM EDTA, pH 8.0, 0.5% Triton X-100, 20% SDS, and 10 mg/mL of proteinase K) and then centrifuged (1200 rpm, 5 min). After extraction with phenol: chloroform: isoamyl alcohol (25:24:1), the obtained pellets were incubated with TE buffer (10 mM Tris-HCl, pH 7.4, 1 mM EDTA, pH 8.0) and RNase A (2 mg/mL, Sigma, St. Louis, MO, USA) for 1 h at 37 °C. Next, electrophoresis was performed on 2% agarose containing ethidium bromide. The DNA bands were measured using a UV Transilluminator Imaging System.

### 2.6. Hoechst Staining Assay

Morphological changes were measured using a Hoechst staining assay by fluorescent microscopy. RC-58T cells were seeded at a concentration of 5 × 10^4^ cells/well, then treated with sanggenol L (10, 20, and 30 µM) for 48 h. After harvesting, the cells were stained with 200 μL of Hoechst 33258 (5 μg/mL) for 10 min at room temperature. This suspension was placed on a glass slide. Cells were measured using a fluorescence microscope (Olympus Optical Co. Ltd., Tokyo, Japan) to determine chromatin condensation and nuclei fragmentation.

### 2.7. Cell Cycle Analysis

Cell cycles were determined by using a Muse™ cell cycle assay kit according to the manufacturer’s protocols. Cells were seeded at a concentration of 5 × 10^4^ cells/well and cultured for 24 h. After culturing, the cells were treated with sanggenol L (10, 20, and 30 µM) for 48 h. The cells were fixed in ice-cold 70% ethanol at 4 °C for 24 h. After resuspension, the cells were added 200 μL of Muse™ cell cycle reagent and incubated for 30 min at room temperature. Cells were analyzed using a Muse™ cell analyzer (Merck KGaA, Darmstadt, Germany).

### 2.8. Western Blot Analysis

The RC-58T cells were seeded at a concentration of 5 × 10^4^ cells/well, then cultured for 24 h. Cells were treated with sanggenol L (10, 20, and 30 µM) for 48 h. The cells were lysed with lysis buffer (1 mM EDTA, 50 mM NaF, 50 mM Tris-HCl, 150 mM NaCl, 30 mM Na4P2O7, 1 mM PMSF, and 2 μg/mL of aprotinin) for 30 min on ice, after which the protein content was determined by using a BCA protein kit (Pierce, Rockford, IL, USA). The samples were separated by 8%–12% SDS-PAGE at 100 V of constant voltage/slab for 90 min. After blocking with 2.5% bovine serum albumin (BSA), the membranes were incubated with primary antibody at 4 °C overnight. Finally, the membranes were treated with secondary antibodies for 1 h at 4 °C. Detection of each protein was demonstrated by using an ECL kit (Santa Cruz Biotechnology, Santa Cruz, CA, USA).

### 2.9. Statistical Analysis

Results were expressed as the percentage of control. Data values were expressed as mean ± SD of triplicate determinations. Statistical analyses were performed by applying one-way analysis of variance (ANOVA) with differences analyzed using Dunnett’s test or Student’s *t*-test. The analyses were conducted using Prism software (GraphPad, La Jolla, CA, USA). Statistical significances are identified by * *p* < 0.05, ** *p* < 0.01, and *** *p* < 0.001.

## 3. Results

### 3.1. Sanggenol L Inhibits Cell Growth in DU145, LNCap, RC-58T, and PC-3 Human Prostate Cancer Cells

Sanggenol L is one of several components of root bark of *M. alba* (Figure 1A). DU145, LNCap, RC-58T, and PC-3 human prostate cancer cells were treated with or without 10, 20, and 30 µM sanggenol L for 48 h and then analyzed by performing SRB assays (Figure 1B). Saggenol L significantly inhibited the growth of RC-58T human prostate cancer cells as well as DU145, LNCap, and PC-3 human prostate cancer cells, whereas RWPE-1 human normal cells derived from prostate epithelial cells were unaffected by sanggenol L treatment. Moreover, sanggenol L induced time- and dose-dependent reductions of cell viability in RC-58T human prostate cancer cells (Figure 1C). Cell morphological changes were evaluated in RC-58T cells upon sanggenol L treatment by using phase-contrast microscopy (Figure 1D). After treatment with various concentrations of sanggenol L for 48 h, cell numbers decreased and cell adhesion was weakened in a dose-dependent manner. These results suggest that sanggenol L treatment can inhibit cell growth in various human prostate cancer cell lines.

### 3.2. Sanggenol L Induces Apoptosis in RC-58T Human Prostate Cancer Cells

**Figure 2:**
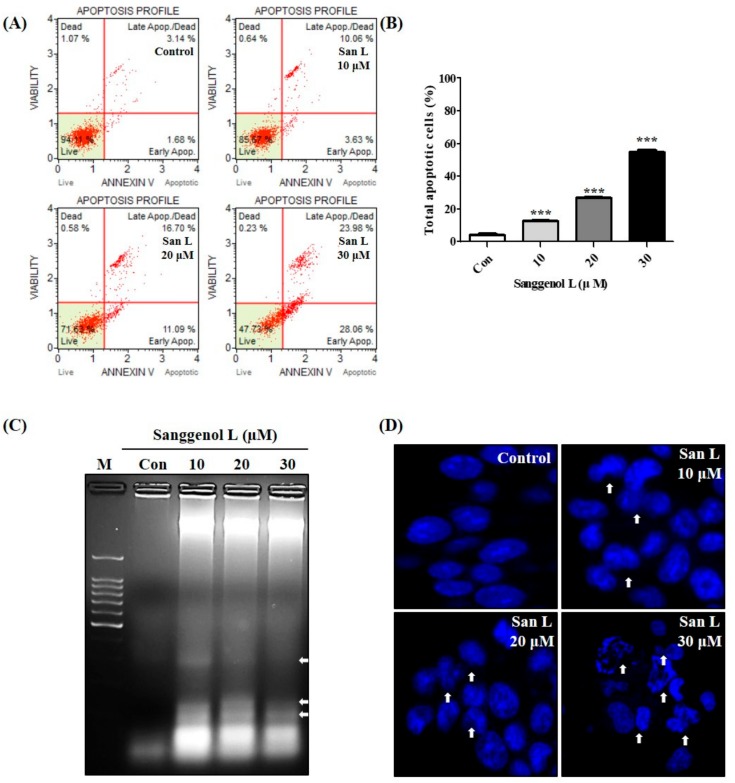
Sanggenol L induces apoptosis in RC-58T human prostate cancer cells. Cells were treated with or without 10, 20, and 30 µM sanggenol L for 48 h. (**A**) Apoptotic cells were evaluated by Annexin V staining assay. (**B**) Total apoptotic cells were quantified and Results were expressed as the percentage of control. Data values were expressed as mean ± SD of triplicate determinations. Significant differences were calculated using Dunnett’s test; *** *p* < 0.001. (**C**) DNA fragmentation was measured by performing 2% agarose gel electrophoresis. (**D**) Nuclear condensations were detected by Hoechst staining assay upon sanggenol L treatment.

We tested whether sanggenol L induces apoptosis in human prostate cancer RC-58T cells by performing Annexin V staining, Hoechst 33,258 staining, and DNA fragmentation assays (Figure 2). RC-58T cells were treated with or without 10, 20, and 30 µM sanggenol L for 48 h. The results showed that apoptotic cell accumulations were significantly enhanced by the sanggenol L treatment (Figure 2A,B). Moreover, as shown in Figure 2C,D, apoptotic biological indicators, including DNA fragmentation, apoptotic bodies, and nuclear condensation were increased by sanggenol L treatment for 48 h. These results clearly show that sanggenol L induces apoptosis in primary prostate cancer RC-58T cells.

### 3.3. Sanggenol L Induces Caspase-Dependent Apoptosis in Prostate Cancer RC-58T Cells

**Figure 3:**
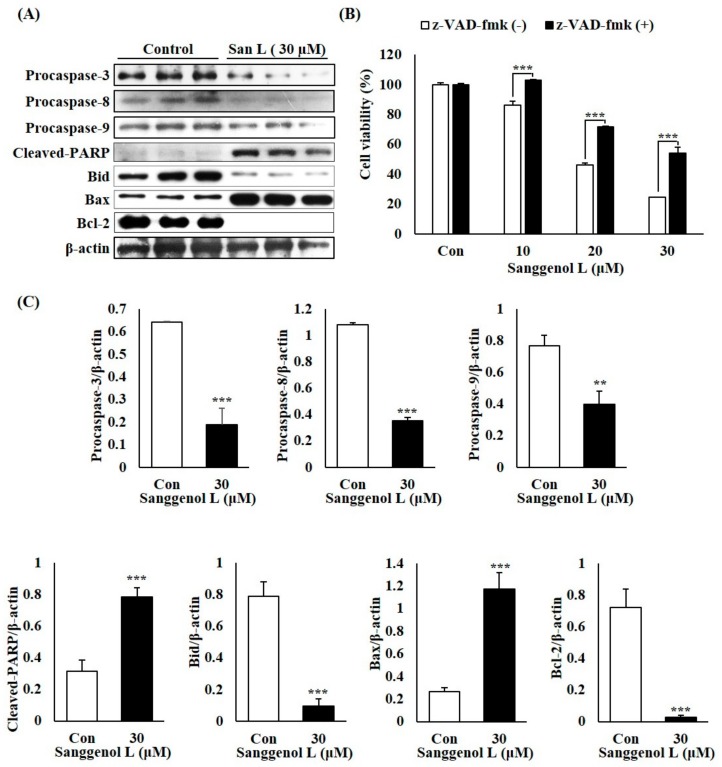
Sanggenol L induces caspase-dependent apoptosis in RC-58T human prostate cancer cells. Cells were treated with or without 30 µM sanggenol L for 48 h. (**A**) The levels of procaspase-3, -8, -9, and cleaved-PARP, Bid, Bax, and Bcl-2 proteins in RC-58T prostate cancer cells. Total cell lysates were subjected to western blot analysis to measure the protein expression levels. (**B**) The influence of z-VAD-fmk (a caspase inhibitor) was measured by SRB assay. Results were expressed as the percentage of control. Data values were expressed as mean ± SD of triplicate determinations. Significant differences were calculated using Dunnett’s test; *** *p* < 0.001. (**C**) Total apoptotic proteins were quantified and data values were expressed as mean ± SD of triplicate determinations. Significant differences were calculated using Student’s *t*-test; ** *p* < 0.01 and *** *p* < 0.001.

To determine whether sanggenol L induces apoptosis through caspase cascades in human prostate cancer cells, we examined levels of procaspases-3, -8, and -9, as well as cleaved-PARP, Bid, Bax, and Bcl-2 by performing western blot analysis and examined the influence of z-VAD-fmk (a caspase inhibitor) using an SRB assay (Figure 3). As shown in Figure 3A,C, the levels of procaspase-3, -8, and -9, as well as Bid and Bcl-2, decreased, while the levels of cleaved-PARP and Bax increased following sanggenol L treatment of prostate cancer RC-58T cells. Furthermore, the caspase inhibitor ‘z-VAD-fmk’ significantly inhibited the sanggenol L-induced cell death in RC-58T cells (Figure 3B). These data show that sanggenol L significantly induced apoptosis via a caspase cascade-dependent pathway in primary prostate cancer RC-58T cells.

### 3.4. Sanggenol L Induces Caspase-Independent Apoptosis in Prostate Cancer RC-58T Cells

**Figure 4:**
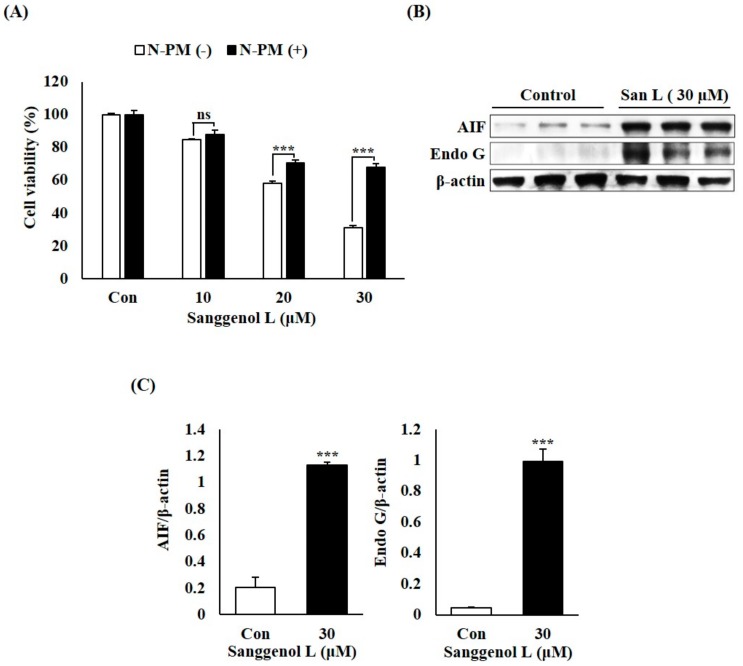
Sanggenol L induces caspase-independent apoptosis through an apoptosis-inducing factor in RC-58T human prostate cancer cells. (**A**) The influence of N-PM (an AIF inhibitor) was measured by SRB assay. Significant differences were calculated using Dunnett’s test; *** *p* < 0.001. (**B**) The levels of AIF and Endo G proteins in RC-58T prostate cancer cells. Total cell lysates were subjected to western blot analysis to measure the protein expression levels. (**C**) Total apoptotic proteins were quantified and data values were expressed as mean ± SD of triplicate determinations. Significant differences were calculated using Student’s *t*-test; *** *p* < 0.001.

To test whether sanggenol L induces apoptotic cell death through the activation of AIF and Endo G in RC-58T cells, we measured the influence of N-PM (AIF inhibitor) by performing SRB assays and determined levels of AIF and Endo G by undertaking western blot analyses (Figure 4). As shown in Figure 4A, AIF inhibitor ‘N-PM’ definitely inhibited sanggenol L-induced apoptotic cell death in RC-58T cells. Furthermore, the levels of AIF and Endo G in prostate cancer RC-58T cells were increased by sanggenol L treatment (Figure 4B,C). These data show that sanggenol L can significantly induce apoptosis through a caspase-independent pathway in primary prostate cancer RC-58T cells.

### 3.5. Sanggenol L Induces Cell Cycle Arrest in RC-58T Human Prostate Cancer Cells

**Figure 5:**
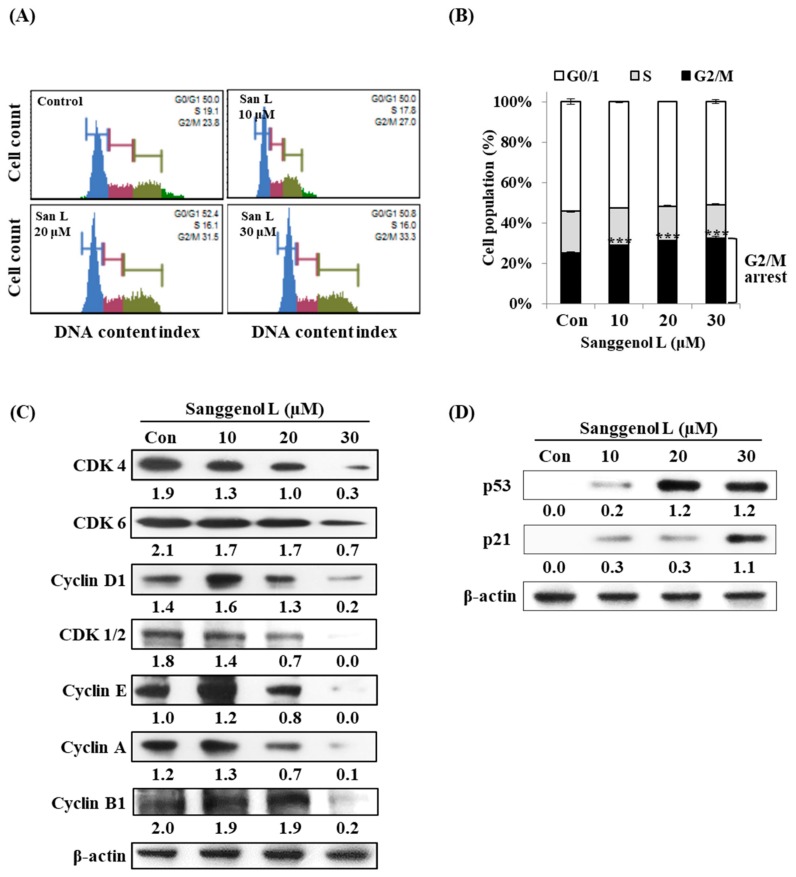
Sanggenol L induces cell cycle arrest in RC-58T cells. (**A**) Cells were treated with sanggenol L (10, 20, and 30 μM) for 48 h and cell cycle progression was analyzed by using the Muse™ cell cycle kit. (**B**) Cell populations at various cell cycle phases were quantified and data values were expressed as mean ± SD of triplicate determinations. Significant differences were calculated using Dunnett’s test; *** *p* < 0.001. (**C**) The levels of cell cycle-related proteins in RC-58T cells. (**D**) The levels of p53 and p21 proteins in RC-58T cells. Total cell lysates were subjected to western blot analysis to determine the protein expression levels. β-Actin, a housekeeping gene, was used as the denominator to quantify relative gene expression levels.

Cancer cells, without a normal cell cycle control process, continue to proliferate [28]. To further investigate whether sanggenol L induces cell cycle arrest in RC-58T prostate cancer cells, we examined the inhibition of cell cycle progression in RC-58T cells by sanggenol L treatment using the Muse™ cell cycle kit (Figure 5). As shown in Figure 2A,B, sanggenol L arrested RC-58T cells in the G2/M phase of the cell cycle. Compared to control cells, the G2/M phase in RC-58T cells was up-regulated by sanggenol L treatment in a dose-dependent manner, whereas G0/G1 and S phases of the cell cycle were unaffected by sanggenol L treatment (Figure 5A,B). Furthermore, we measured levels of cell cycle-related proteins in RC-58T cells following sanggenol L treatment (Figure 5C,D). Treatment with sanggenol L resulted in down-regulation of CDK1/2, CDK4, CDK6, cyclin D1, cyclin E, cyclin A, and cyclin B1, and up-regulation of p53 and p21 proteins in dose-dependent manners. These results demonstrate that sanggenol L can inhibit cell proliferation of RC-58T cells through cell cycle arrest, especially at the G2/M phase.

### 3.6. Sanggenol L Inhibits Cell Growth of Prostate Cancer Cells Through Suppression of the PI3K/Akt/mTOR Signaling Pathway

**Figure 6:**
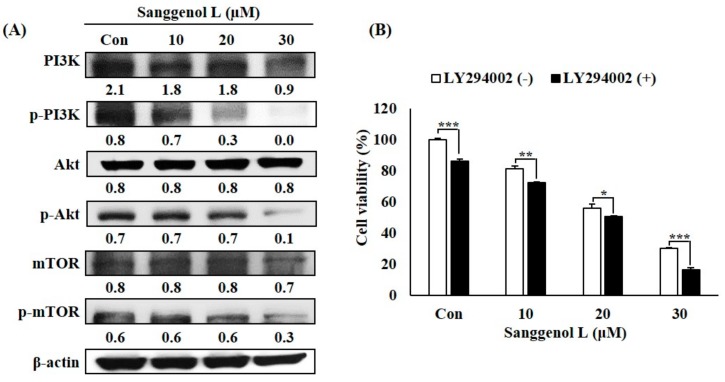
Sanggenol L inhibits cell growth of prostate cancer cells through suppression of the PI3K/Akt/mTOR signaling pathway. (**A**) The levels of phospho-PI3K, phospho-Akt, and phospho-mTOR proteins in RC-58T prostate cancer cells. Cells were treated with or without 10, 20, and 30 μM sanggenol L for 48 h. Total cell lysates were subjected to western blot analysis to measure the protein expression levels. β-Actin, a housekeeping gene, was used as the denominator to quantify relative gene expression levels. (**B**) The influence of LY294002 (a PI3K inhibitor) was determined by SRB assay. Results were expressed as the percentage of control. Data values were expressed as mean ± SD of triplicate determinations. Significant differences were calculated using Student’s *t*-test; * *p* < 0.05, ** *p* < 0.01, and *** *p* < 0.001.

We evaluated whether expression levels of the phospho-PI3K, phospho-Akt, and phospho-mTOR signaling pathways were associated with sanggenol L treatment in RC-58T cells (Figure 6). The levels of phospho-PI3K, phospho-Akt, and phospho-mTOR decreased following exposure to sanggenol L treatment in RC-58T cells (Figure 6A). Furthermore, pre-treatment of RC-58T cells with a PI3K inhibitor followed by treatment with sanggenol L resulted in greater inhibition of cell proliferation compared to that in non-PI3K-inhibitor-treated cells (Figure 6B). These results suggest that the inhibition of the PI3K/Akt/mTOR signaling pathway is associated with the saggenol L-induced cell growth inhibition in primary prostate cancer RC-58T cells.

## 4. Discussion

Sanggenol L is one of the reported effective components of *Morus alba* root bark [17] and has been reported to have pharmacological activities, such as neuroprotective and cytotoxic effects [29,30]. Sanggenol L was also reported to induce apoptosis via activation of caspase and inhibition of NF-kB signaling in ovarian cancer cells [18]. However, the mechanism of the cell death-inducing effect of sanggenol L in prostate cancer cells remains unclear. As shown in Figure 1, sanggenol L treatment can inhibit cell growth in various human prostate cancer cells, including RC-58T, LNCap., DU145, and PC-3, whereas RWPE-1, a normal prostate cell line, was unaffected by sanggenol L treatment.

Apoptosis is also known as programmed cell death, and it has specific morphological features, such as accumulation of cells at the sub-G1 phase of the cell cycle, DNA fragmentation, chromatin condensation, and formation of apoptotic bodies, and it is associated with the regulation of caspase cascades [19]. Based on the evidence of induction of apoptosis in various cancer cell types, many natural products have been shown to have anticancer properties [11,12,22]. As shown in Figure 2, apoptotic cell accumulations were significantly increased with sanggenol L treatment in RC-58T cells. Moreover, apoptotic biological markers including DNA fragmentation, apoptotic bodies, and nuclear condensation were increased by sanggenol L treatment in RC-58T cells. We also tested whether sanggenol L induces apoptosis in human prostate cancer PC-3 cells by performing Annexin V staining (Supplementary Table S1). Apoptotic cell accumulations were significantly enhanced in PC-3 cells by the sanggenol L treatment. These results suggest that sanggenol L clearly induces apoptosis in both RC-58T primary prostate cancer cells and PC-3 cells.

Apoptosis is related to the regulation of caspase cascades, and caspases are known as effector (caspases-3, -6, -7) or initiator (extrinsic pathway; caspases-2, -8, -10, and intrinsic pathway; caspase-9) caspases. When caspases are activated, the procaspase levels decrease via the formation of cleaved-caspases [31]. Along with these caspase activities, apoptosis is also regulated by apoptosis-related proteins, also known as Bcl-2 family members, such as Bid, Bax, and Bcl-2, [32]. In this study, we showed that the levels of procaspases-3, -8, and -9, Bid, and Bcl-2 decreased and the levels of cleaved-PARP and Bax increased in prostate cancer RC-58T cells following sanggenol L treatment (Figure 3A,C). Moreover, the caspase inhibitor ‘z-VAD-fmk’ significantly inhibited the sanggenol L-induced cell death in RC-58T cells (Figure 3B). These results indicate that sanggenol L can induce apoptosis via a caspase -dependent pathway in primary prostate cancer RC-58T cells (Figure 3).

Apoptosis is associated not only with regulation of the caspase-dependent pathway but also with regulation of the caspase-independent pathway [24]. AIF and Endo G are representative caspase-independent pathway components and are released from mitochondria to the cytosol [24]. Several previous studies have shown that during apoptotic cell death, mitochondrial membrane permeabilization leads to the release of AIF and Endo G, the loss of mitochondrial function, and DNA degradation [11,22,33]. In this study, the levels of AIF and Endo G in prostate cancer RC-58T cells were increased by sanggenol L treatment (Figure 4B,C). Moreover, the AIF inhibitor ‘N-PM’ notably inhibited sanggenol L-induced apoptotic cell death in RC-58T cells (Figure 4A). These results indicate that sanggenol L can induce apoptosis in primary prostate cancer RC-58T cells through the caspase-independent pathway (Figure 4).

Cell cycle deregulation is a common feature of human cancers, and cancer cells, which do not have a normal cell cycle control process, continue to proliferate [28]. CDK/Cyclins form a family of heterodimeric kinases that have key roles in the regulation of cell cycle progression and other major biological processes [34]. CDK/Cyclins are divided into regulators of different cell cycle phases: G0/G1 phase—cyclin D and CDK4/6; S phase—cyclin E and CDK2; and G2/M phase—cyclin A, cyclin B1, and CDK1/2 [34]. In this paper, sanggenol L inhibited the cell cycle G2/M phase in RC-58T cells (Figure 5). G2/M phase accumulation in RC-58T cells was up-regulated by sanggenol L treatment in a dose-dependent manner, whereas the populations of cells in G0/G1 and S phase were unaffected by sanggenol L treatment (Figure 5A,B). Moreover, treatment with sanggenol L resulted in down-regulation of CDK1/2, CDK4, CDK6, cyclin D1, cyclin E, cyclin A, and cyclin B1 proteins in dose-dependent manners (Figure 5C). Although the cell cycle assessment only identified G2/M phases arrest, overall, the cell cycle-related proteins decreased following exposure to sanggenol L (Figure 5). Furthermore, treatment with sanggenol L resulted in up-regulation of p53 and p21 proteins, which are tumor suppression proteins associated with the cell cycle (Figure 5D). These results suggest that sanggenol L can inhibit cell proliferation of RC-58T cells through cell cycle arrest, especially arrest at G2/M phase and up-regulation of p53 and p21 (Figure 5). We also examined the effect of sanggenol L treatment on cell cycle progression in PC-3 cells using the Muse™ cell cycle kit (Supplementary Table S2). Sanggenol L inhibited the cell cycle progression in PC-3 cells. The G2/M phase in PC-3 cells was up-regulated by 30 μM sanggenol L treatment. These results demonstrate that sanggenol L can inhibit cell proliferation of RC-58T primary prostate cancer cells and PC-3 cells through cell cycle arrest.

Phosphorylation of PI3K activates the phosphorylation of Akt and mTOR, which have central roles in the regulation of cell proliferation, growth, and survival [35]. Many previous studies have shown that natural substances have the ability to suppress the PI3K/Akt/mTOR signaling pathway in several cancer cells, thus, targeting the PI3K/Akt/mTOR signaling pathway is a key strategy for the suppression of cancer [36,37,38]. The levels of phospho-PI3K, phospho-Akt, and phospho-mTOR decreased in RC-58T cells following exposure to sanggenol L treatment (Figure 6A). Furthermore, pre-treatment of RC-58T cells with a PI3K inhibitor resulted in marked inhibition of cell proliferation compared to that in non-PI3K-inhibitor-treated cells (Figure 6B). These results suggest that the PI3K/Akt/mTOR signaling pathway is associated with the observed sanggenol L-induced cell growth inhibition in primary prostate cancer RC-58T cells (Figure 6).

In this study, we provide evidence that exposure to sanggenol L can suppress cell growth in prostate cancer cells by triggering caspase-dependent and caspase-independent apoptosis, as well as cell cycle arrest, via the activation of tumor suppressor p53 and inhibition of PI3K/Akt/mTOR signaling. This study is the first to show that apoptosis and cell cycle arrest in human prostate cancer cells can be induced by sanggenol L. More studies are required, including in vivo animal studies, to further examine whether sanggenol L treatment is effective against prostate cancer. Human studies are required to evaluate whether sanggenol L has a potential to become a useful chemotherapeutic agent against human prostate cancer.

## Supplementary Materials

The following are available online at https://www.mdpi.com/2072-6643/12/2/488/s1, Table S1: Sanggenol L induces apoptosis in PC-3 human prostate cancer cells, Table S2: Sanggenol L induces cell cycle arrest in PC-3 human prostate cancer cells.
