# Supplementary material for: Sanggenol L Induces Apoptosis and Cell Cycle Arrest via Activation of p53 and Suppression of PI3K/Akt/mTOR Signaling in Human Prostate Cancer Cells
Source: Nutrients. 2020 Feb 14;12(2):488. doi: 10.3390/nu12020488 (PMC7071324; doi:10.3390/nu12020488)
Supplement: Supplementary file 1 [file nutrients-12-00488-s001.zip › nutrients-677057-supplementary/Supplementary Table S1.docx]

**
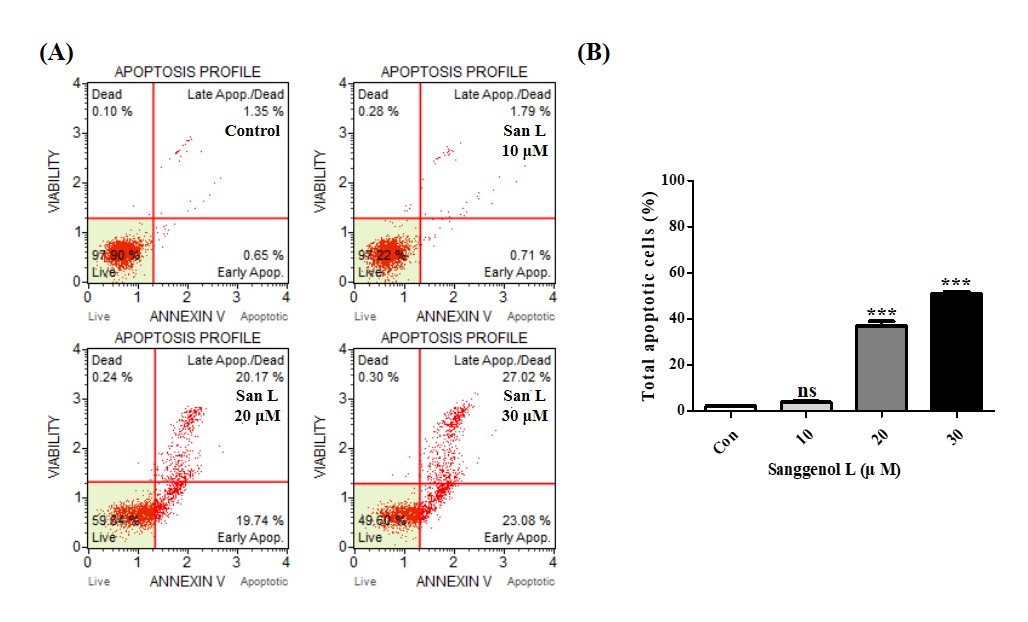
**

**Supplementary Table S1:** Sanggenol L induces apoptosis in PC-3 human prostate cancer cells. Cells were treated with or without 10, 20, and 30 µM sanggenol L for 48 h. (A) Apoptotic cells were evaluated by Annexin V staining assay. (B) Total apoptotic cells were quantified and results were expressed as the percentage of control. Data values were expressed as mean ± SD of triplicate determinations. Significant differences were calculated using Dunnett’s test; *** *p < 0.001*.
