# Supplementary material for: Sanggenol L Induces Apoptosis and Cell Cycle Arrest via Activation of p53 and Suppression of PI3K/Akt/mTOR Signaling in Human Prostate Cancer Cells
Source: Nutrients. 2020 Feb 14;12(2):488. doi: 10.3390/nu12020488 (PMC7071324; doi:10.3390/nu12020488)
Supplement: Supplementary file 1 [file nutrients-12-00488-s001.zip › nutrients-677057-supplementary/Supplementary Table S2.docx]

**
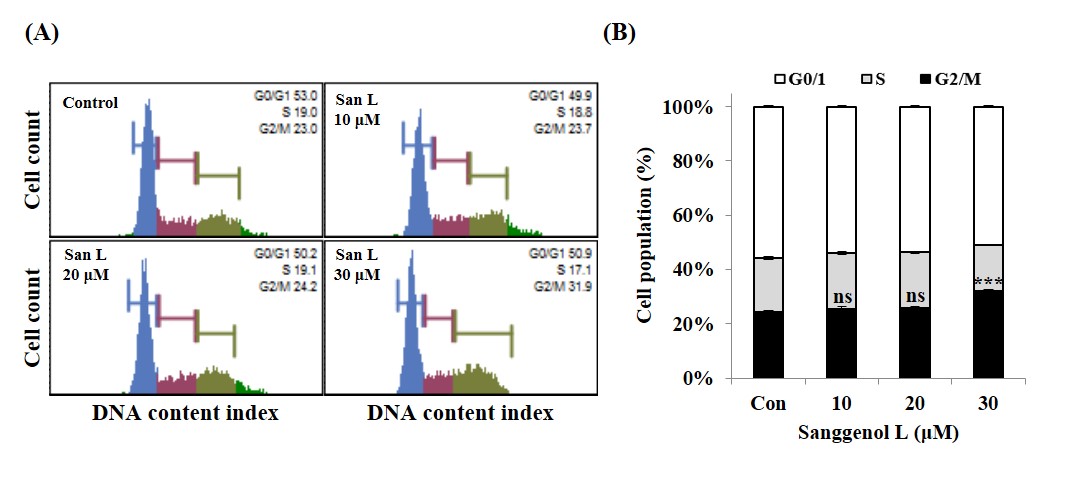
**

**Supplementary Table S2:** Sanggenol L induces cell cycle arrest in PC-3 human prostate cancer cells. (A and B) Cells were treated with sanggenol L for 48 h and cell cycle progression was analyzed by using the Muse™ cell cycle kit. Cell populations at various cell cycle phases were quantified and data values were expressed as mean ± SD of triplicate determinations. Significant differences were calculated using Dunnett’s test; *** *p < 0.001*.
